# Genome-Wide Identification of the ZmNF-Y Transcription Factor Family in Maize and Functional Characterization of ZmNF-YA7 in Drought Tolerance

**DOI:** 10.3390/ijms27188319

**Published:** 2026-09-18

**Authors:** Doudou Sun, Yichen Jin, Mengru Liu, Xuan Zhang, Tongtong Hao, Xiaolin Wu, Jinghua Zhang

**Affiliations:** 1 State Key Laboratory of High-Efficiency Production of Wheat-Maize Double Cropping, College of Life Sciences, Henan Agricultural University, Zhengzhou 450046, China; sundd@henau.edu.cn (D.S.);; 2 Postdoctoral Station of Crop Science, Henan Agricultural University, Zhengzhou 450046, China

**Keywords:** NF-Y transcription factor, ZmNF-YA7, antioxidant enzymes, xylem vessel, drought tolerance

## Abstract

The nuclear factor Y (NF-Y) transcription factor family plays crucial roles in plant growth, development, and stress responses. However, studies on the NF-Y family in maize remain relatively limited. In this study, we identified 53 *NF-Ys* in the maize genome, which were classified into *ZmNF-YA*, *ZmNF-YB*, and *ZmNF-YC* subfamilies. These genes exhibited diverse physicochemical properties and were unevenly distributed across the 10 chromosomes. Synteny analysis revealed 48 collinear gene pairs between maize and rice, indicating close evolutionary relationships. Gene structure and conserved motif analyses showed both conservation and divergence within the family. Promoter analysis identified abundant cis-acting elements related to hormone response, abiotic stress, light response, and growth regulation. Expression profiling under drought stress revealed that *ZmNF-Ys* respond differentially, with some showing strong induction. Functional characterization demonstrated that ZmNF-YA7 positively regulates drought tolerance in maize. Overexpression of *ZmNF-YA7* enhanced drought tolerance through multiple mechanisms: reducing oxidative damage by increasing antioxidant enzyme activities, accumulating osmoprotectants, and modulating xylem vessel anatomy (larger diameter and lower density under drought). These findings provide comprehensive insights into the *ZmNF-Y* gene family and highlight ZmNF-YA7 as a promising candidate for improving drought tolerance in maize breeding programs.

## 1. Introduction

Transcription factors regulate gene expression by recognizing and binding to specific DNA elements in the promoter regions of target genes, thereby playing diverse and essential roles in growth and development in plants [1,2]. Nuclear Factor Y (NF-Y), also known as Heme Activator Protein (HAP) or CCAAT-Box Binding Factor (CBF), is a transcription factor widely present in eukaryotes [3]. The NF-Y complex consists of three distinct subunits: NF-YA (HAP2/CBF-B), NF-YB (HAP3/CBF-A), and NF-YC (HAP5/CBF-C) [4]. In animals and yeast, each NF-Y subunit is encoded by a single gene. In plants, however, each subunit is not solitary; rather, each subfamily has evolved multiple members that function by forming heterodimers or heterotrimers [5]. Due to the vast number of potential dimeric or trimeric combinations, many NF-Y gene families in plants may exhibit functional redundancy. In recent years, numerous studies have demonstrated that NF-Y performs diverse biological functions during plant growth and development, including embryogenesis [6], seed germination [7], flowering-time regulation [8,9,10], photomorphogenesis [11], fruit ripening [12], and responses to abiotic and biotic stresses [13,14,15,16,17].

NF-YA proteins are typically localized in the nucleus. Most NF-YA proteins are capable of binding to the CCAAT box in the promoter regions of target genes, albeit with varying binding affinities [5,18]. NF-YA proteins all possess a conserved domain composed of approximately 56 amino acids, which consists of two α-helices (α1 and α2). The α1 helix, located at the N-terminus, interacts with the NF-YB and NF-YC subunits, while the α2 helix, located at the C-terminus, specifically binds to the CCAAT box [3,5].

NF-YB contains a histone-fold domain (HFD) structurally similar to that of histone H2B [5]. The HFD typically consists of at least three α-helices separated by two loop structures [19]. The α1 helix of NF-YB carries a putative DNA-binding domain. Unlike NF-YA and NF-YC proteins, the NF-YB subunit lacks a nuclear localization signal (NLS). Therefore, the formation of the NF-YB/YC heterodimer is essential for the translocation of NF-YB from the cytoplasm to the nucleus [20,21].

NF-YC possesses a HFD structurally analogous to that of histone H2A, also composed of three α-helices. The α1 helix of NF-YC primarily stabilizes and interacts with the DNA backbone, wrapping around the CCAAT box [18]. Additionally, the α1 helix contains conserved amino acid residues that are recognized as part of the DNA-binding domain [3].

Abiotic stress represents one of the most pervasive environmental factors affecting crop productivity [22,23]. Plants grow in soil, where changes in soil water availability are a common abiotic stress they face [24,25]. Transcriptomic analysis under drought conditions revealed that a large number of *NF-Y* genes in Arabidopsis are responsive to stress, with most *NF-YA* genes showing up-regulated expression [20].

NF-Ys interact with miR169 to co-regulate plant drought responses [26]. In maize, the expression of most miR169s is down-regulated under PEG-induced drought stress, while the expression of their target gene *ZmNF-YA* varies with treatment duration, showing distinct expression patterns [27]. In Arabidopsis, overexpression of miR169a increases drought sensitivity by repressing the expression of *AtNF-YA5* [28,29]. Under drought conditions, high expression of AtNF-YA5 not only affects stomatal closure and promotes water transport in vascular tissues, but also participates in the ABA pathway to further activate drought-responsive gene expression [30]. It was found that the miR169z-NF-YA5-GPDHc1 module enhances drought tolerance by increasing NAD^+^ levels to inhibit Reactive Oxygen Species (ROS) production in *Populus* [31].

Under drought stress, poplar PdNF-YB21 interacts with PdFUS3 to co-activate *NINE-CIS-EPOXYCAROTENOID DIOXYGENASE 3 (PdNCED3)* expression, promoting ABA biosynthesis and auxin transport in roots. This enhances root growth and increases lignin content, thereby improving poplar drought tolerance [32]. AtNF-YB2 interacts with DREB2A to activate drought-responsive gene expression and improve plant drought tolerance [33]. Overexpression of maize *ZmNF-YB2*, wheat *TaNF-YB2*, barley *HvNF-YB7*, poplar *PdNF-YB7* and spruce *PwNF-YB3* all enhance drought tolerance [24,34,35,36], demonstrating that the NF-Y-mediated drought response mechanism is conserved across species.

In recent years, the functions of several *NF-Y* genes in drought resistance have been extensively characterized in major crops. ZmNF-YA3 can bind to the promoter regions of *bHLH92*, *FAMA*, and *MYC4*, thereby enhancing drought tolerance and thermotolerance [37]. Overexpression of *ZmNF-YB2* significantly enhances drought tolerance and improves grain yield under water-deficit conditions in maize [24]. Overexpression of *ZmNF-YB16* boosts photosynthetic efficiency and antioxidant capacity, thereby increasing both yield and drought resilience [38]. Overexpression of *ZmNF-YA1* improved drought and salt stress tolerance and root development of maize, whereas *zmnf-ya1* mutants exhibited drought and salt stress sensitivity [39]. In wheat, overexpression of *TaNF-YB4* under well-irrigated conditions substantially increases spike numbers and raises grain yield by 20–30% [40]. In rice, *OsNF-YA7* is induced by drought stress and participates in drought responses through an ABA-independent manner [41]. Rice overexpressing *OsNF-YB12* was more sensitive to salinity and PEG osmotic stresses at seed germination stage, as well as reduced drought tolerance at seedling stage [42].

NF-Ys recognize and bind to the CCAAT box, a cis-element present in the promoter regions of approximately 20–30% of eukaryotic genes. Compared with animals and fungi, the *NF-Y* gene family in plants has undergone significant duplication and expansion, resulting in a variable number of gene family members across plant species. *NF-Ys* participate in nearly all stages of plant growth and development, as well as in responses to abiotic and biotic stresses. To date, *NF-Y* gene family members have been identified in numerous plant species, yet the functions of most members remain poorly characterized. Studies on the *NF-Y* family in maize remain relatively limited.

In this study, we identified 53 *NF-Ys* in the maize genome classified into *ZmNF-YA*, *ZmNF-YB* and *ZmNF-YC* subfamilies. These genes exhibited diverse physicochemical properties and promoter analysis identified abundant cis-acting elements related to abiotic stress and growth regulation. Expression profiling under drought stress revealed that *ZmNF-Ys* respond differentially, with some showing strong induction. Overexpression of *ZmNF-YA7* enhanced drought tolerance through multiple mechanisms: reducing oxidative damage by increasing antioxidant enzyme activities, accumulating osmoprotectants, and modulating xylem vessel anatomy (larger diameter and lower density under drought). Elucidating the molecular regulatory network of NF-Y transcription factors is of great significance for leveraging their characteristics in crop genetic improvement and sustainable agricultural production.

## 2. Results

### 2.1. Identification and Physicochemical Characterization of the ZmNF-Y Gene Family Members

As shown in Table 1, a total of 53 ZmNF-Ys with typical domains were identified. The amino acid (aa) lengths ranged from 91 aa (Zm00001eb065670) to 367 aa (Zm00001eb064730). The protein molecular weights (MW) ranged from 10,394.6 Da (Zm00001eb065670) to 39,341.3 Da (Zm00001eb064730). The theoretical isoelectric points (pI) ranged from 4.62 (Zm00001eb302780) to 11.11 (Zm00001eb216950). Nineteen proteins with pI above 7 were classified as basic proteins, while the rest were acidic. The instability indices ranged from 31.49 to 84.05, with values exceeding 40 indicating unstable proteins. The grand average of hydropathicity (GRAVY) values for all proteins were negative, indicating that all ZmNF-Ys are hydrophilic. Subcellular localization predictions showed that most members of the ZmNF-YA and ZmNF-YC subfamilies are localized in the nucleus, while Zm00001eb316260, Zm00001eb274340, and Zm00001eb413100 are predicted to be localized in the cytoplasm; and the members of ZmNF-YB subfamily are localized in the cytoplasm.

### 2.2. Chromosomal Localization of the ZmNF-Y Gene Family

**Figure 1:**
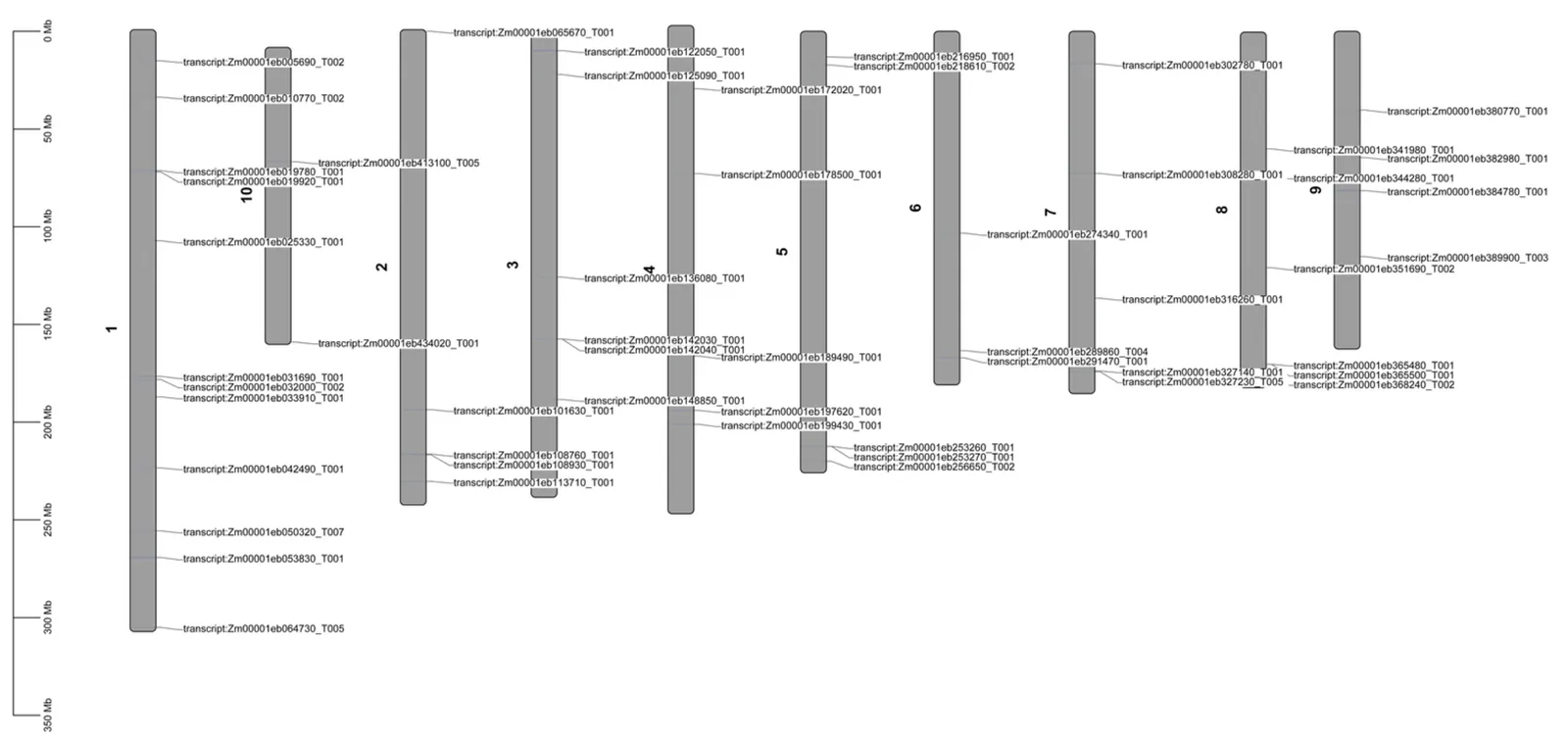
Chromosomal distribution of the *ZmNF-Ys*. 1–10 indicate maize chromosome IDs, and the labels on each chromosome represent the transcript IDs of *ZmNF-Y* family members.

The results of chromosomal localization analysis for the *ZmNF-Y* gene family members indicated that these genes are not randomly distributed across the chromosomes. Chromosome 1 contains the highest number of *ZmNF-Ys*, with a total of 12; chromosomes 2, 4, 5, and 7 each harbor 5 genes; chromosomes 3 and 8 each contain 6 genes, while the remaining chromosomes show varying gene counts: chromosome 6 has 3 genes, chromosome 9 has 4 genes, and chromosome 10 has 2 genes (Figure 1).

### 2.3. Phylogenetic Analysis of the ZmNF-Y Gene Family

**Figure 2:**
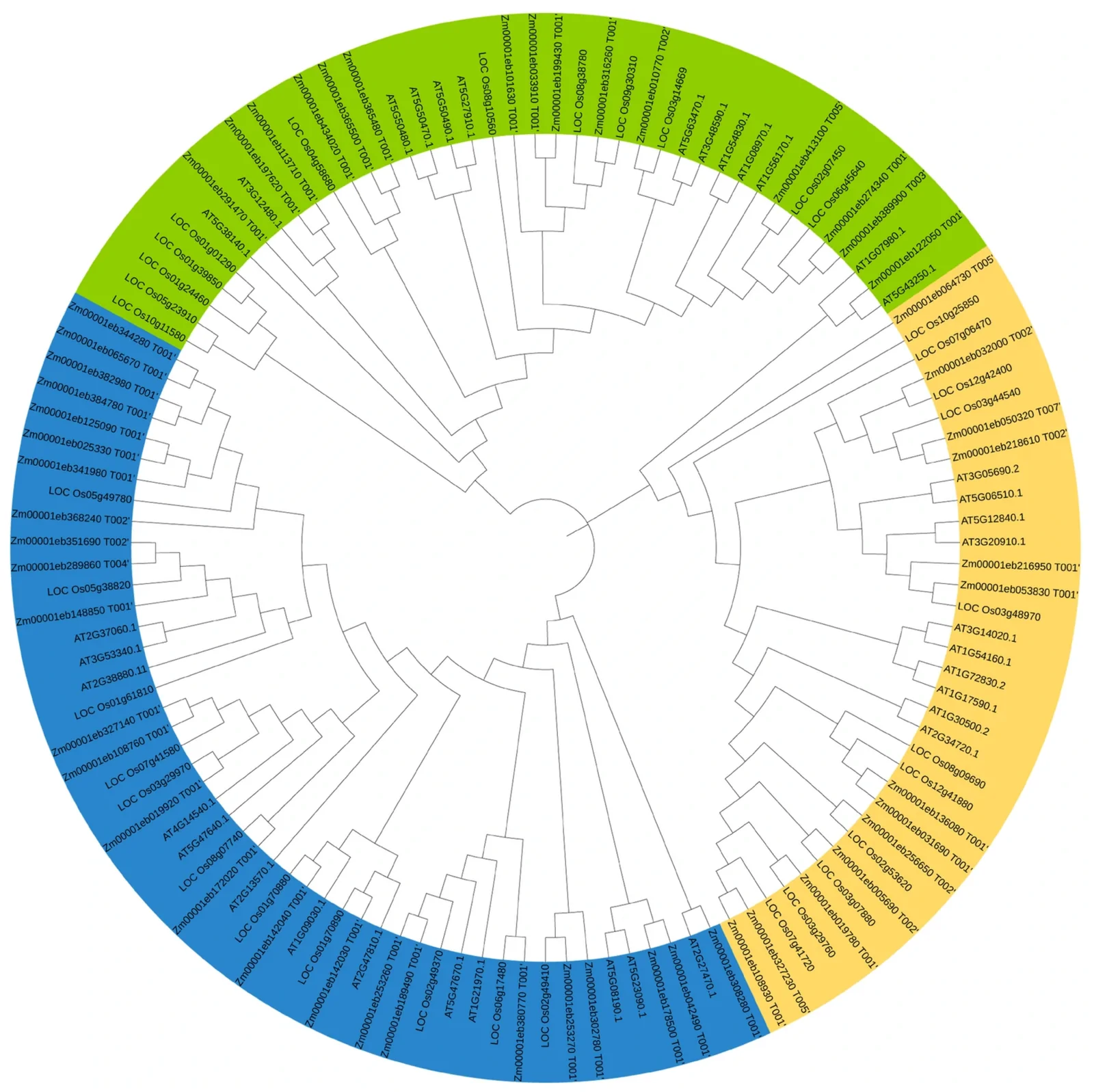
Phylogenetic tree of the ZmNF-Ys. Yellow represents the ZmNF-YA subfamily; blue represents the ZmNF-YB subfamily; and green represents the ZmNF-YC subfamily. The tree was built with MUSCLE v5.1 alignment (default parameters) + IQ-TREE 2.4.0 (ModelFinder, best-fit model JTT + F + R4, 1000 ultrafast bootstraps) (Supplementary Table S1).

Using NF-Y protein sequences from Arabidopsis and rice as references, the phylogenetic relationships of the ZmNF-Y family were further clarified. A phylogenetic tree was constructed using a total of 123 NF-Y protein sequences from Arabidopsis, maize, and rice (Figure 2). The results showed that the *ZmNF-Y* family can be evolutionarily divided into three subfamilies: ZmNF-YA, ZmNF-YB, and ZmNF-YC, containing 13, 25, and 15 members, respectively.

### 2.4. Synteny Analysis of the ZmNF-Ys

**Figure 3:**
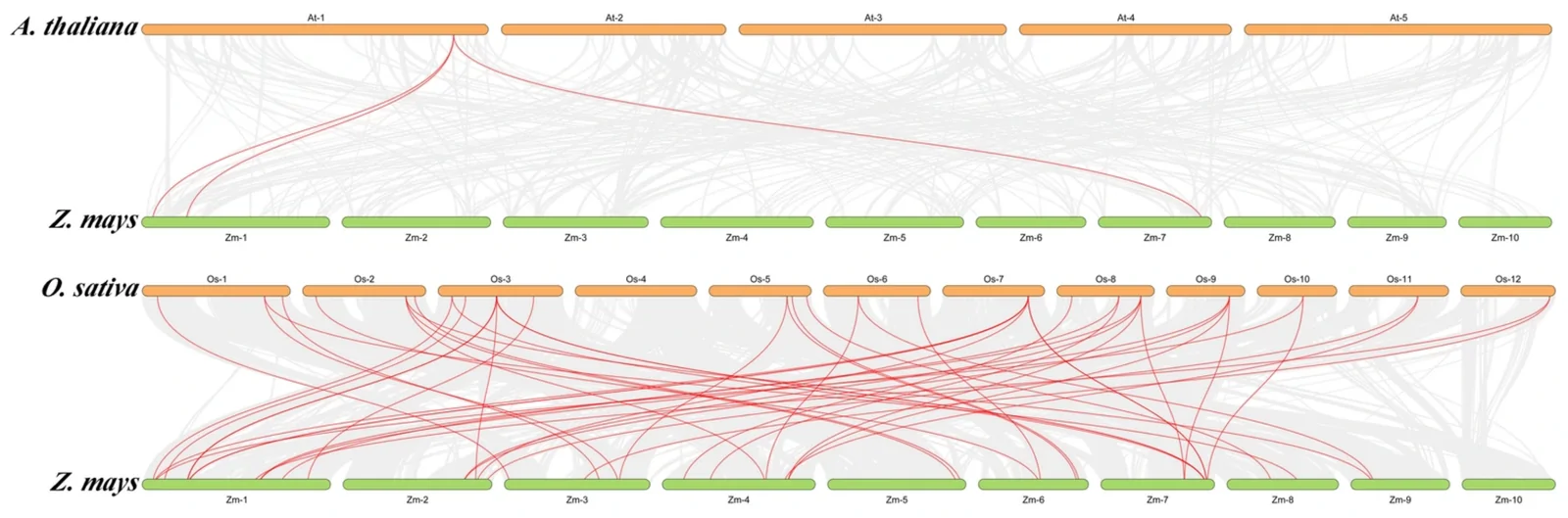
Collinearity analysis of *NF-Ys* in maize, Arabidopsis, and rice. Red lines between chromosomes indicate gene pairs with segmental duplications, and gray lines represent background collinear blocks across the whole genome.

To elucidate the evolutionary relationships of the *NF-Y* gene family, we constructed synteny maps between maize and Arabidopsis, as well as between maize and rice. Three syntenic gene pairs were identified between maize and Arabidopsis (Figure 3), while 48 syntenic gene pairs were identified between maize and rice (Figure 3). The Supplementary Table S2 lists, for each *ZmNF-Y* member: gene ID; the paired syntenic ortholog ID in rice/Arabidopsis. All syntenic pairs were identified with MCScanX-Super Fast plugin implemented in TBtools v2.376. The built-in BLAST module was used with default parameters and an e-value ≤ 1 × 10^−10^) and manually inspected.

**Figure 4:**
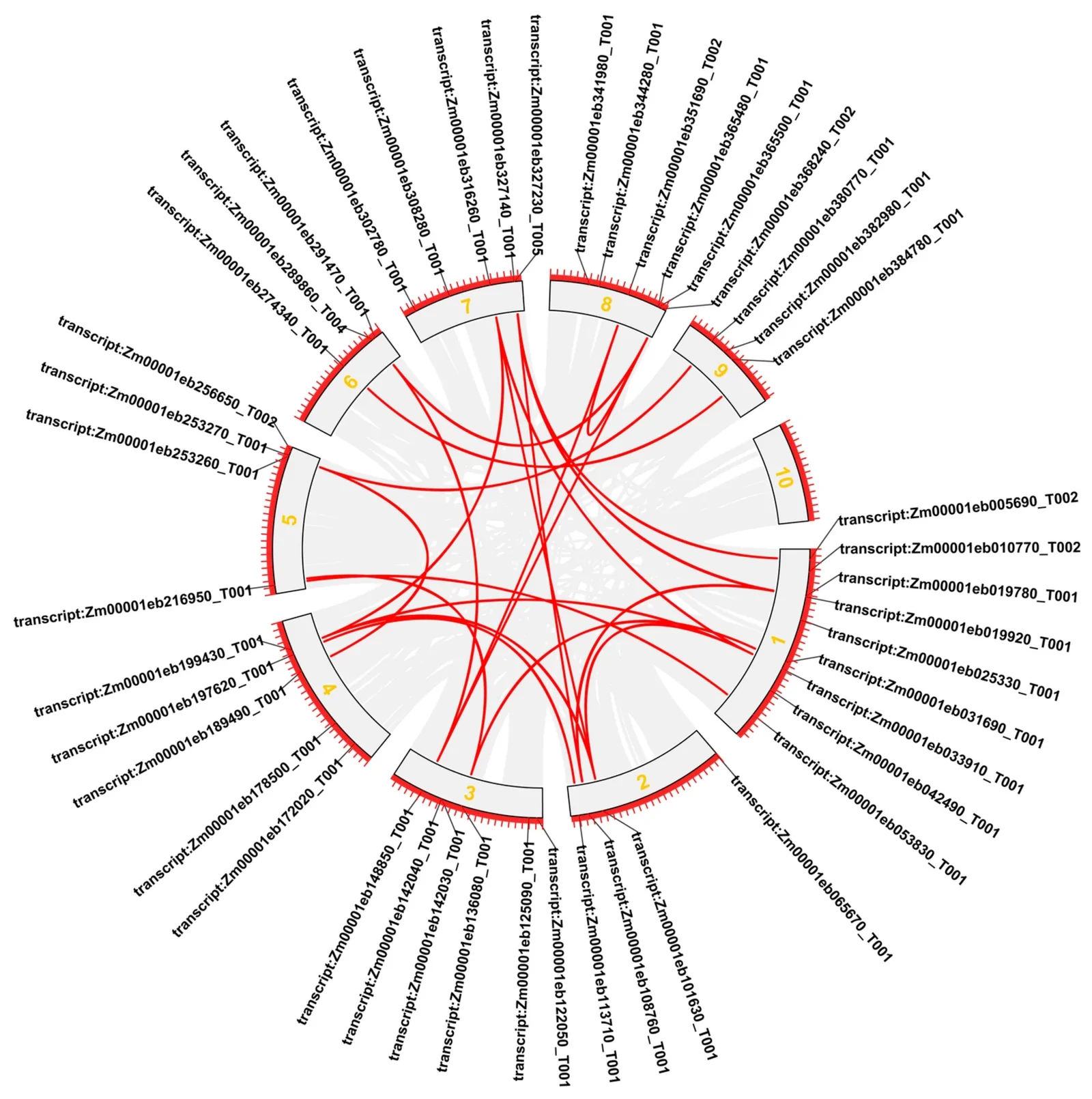
Collinearity analysis of *ZmNF-Ys*. Numbers on the outermost circle represent the ten maize chromosomes (1 to 10). Heat maps and histograms along the rectangles represent gene densities on chromosomes. Gray lines indicate syntenic blocks in the maize genome, and red lines between chromosomes indicate gene pairs with segmental duplications.

The results of intra-species synteny analysis revealed that a total of 45 gene pairs are distributed across the 10 chromosomes of maize (Figure 4). Chromosome 1 harbors the highest number of gene pairs, with 9, while no gene pairs are located on chromosome 10. Specifically, gene synteny was observed both within localized chromosomal regions and across distant chromosomal segments, reflecting multiple events of gene duplication and segmental rearrangement during the evolution of *ZmNF-Ys*.

### 2.5. Analysis of Gene Structure and Conserved Motifs in the ZmNF-Ys

**Figure 5:**
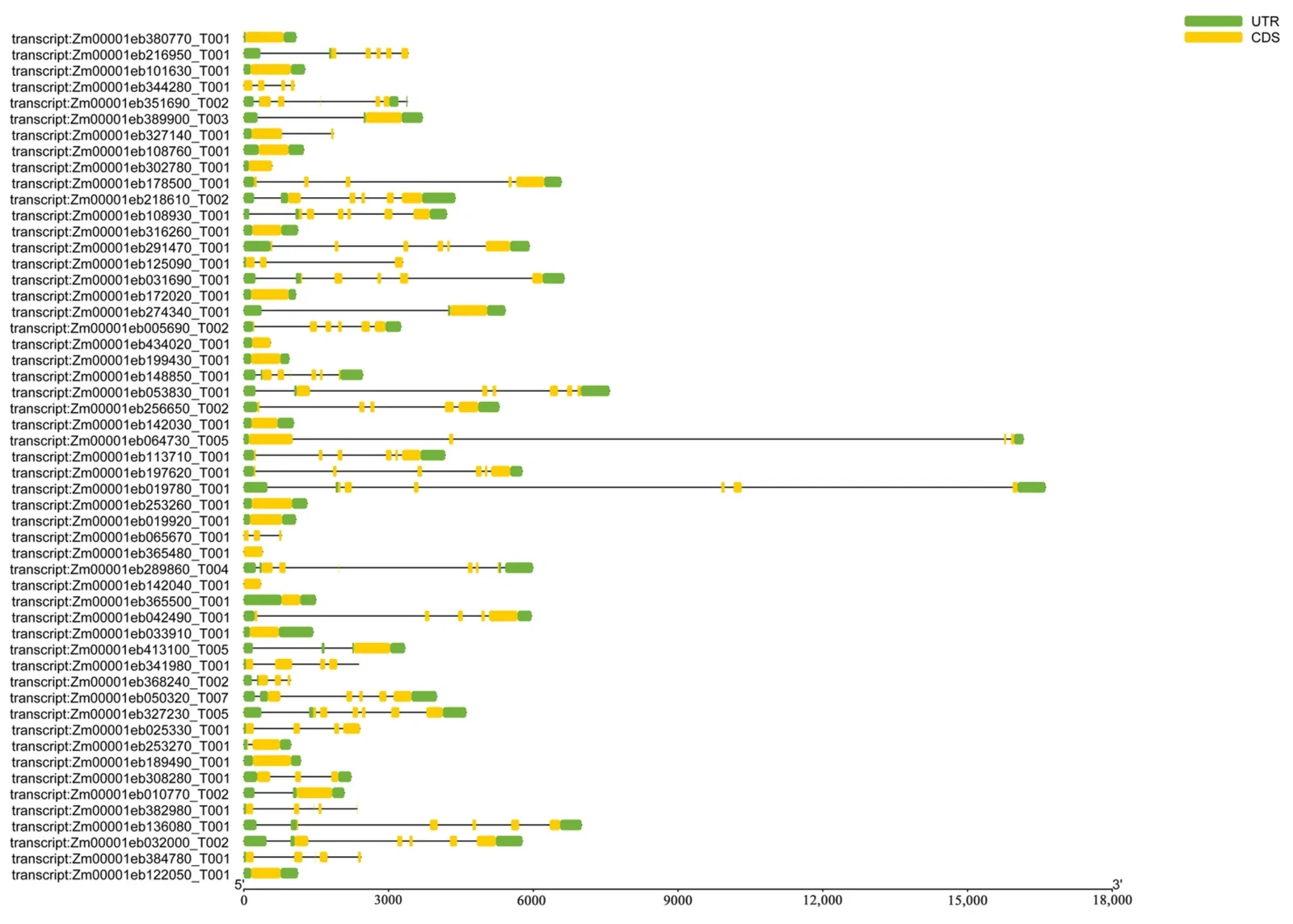
Gene structure analysis of *ZmNF-Ys*. Green bars represent untranslated regions (UTRs), and yellow bars represent coding sequences (CDSs). The scale bar at the bottom indicates gene length (bp).

To elucidate the gene structure characteristics of *ZmNF-Y* gene family members, a visual analysis of the distribution of coding sequences (CDSs) and untranslated regions (UTRs) was conducted (Figure 5). Significant differences in gene structure were observed among members of the *ZmNF-Y* gene family. In terms of gene length, the full-length genes spanned a wide range, with Zm00001eb064730 and Zm00001eb019780 exceeding 15,000 bp, while sequences such as Zm00001eb142040 were shorter, less than 2000 bp, reflecting substantial variation in sequence length within the family. Differences were also noted in the distribution of CDSs and UTRs. Most genes contained both CDSs and UTRs; however, Zm00001eb344280, Zm00001eb065670, Zm00001eb365480, and Zm00001eb142040 comprised only CDSs without UTRs. A majority of the *ZmNF-Ys* contained multiple CDS fragments, with varying spacer lengths between them. Some genes exhibited numerous and widely distributed CDS fragments, whereas others displayed relatively continuous CDS regions, indicating structural complexity within this gene family.

**Figure 6:**
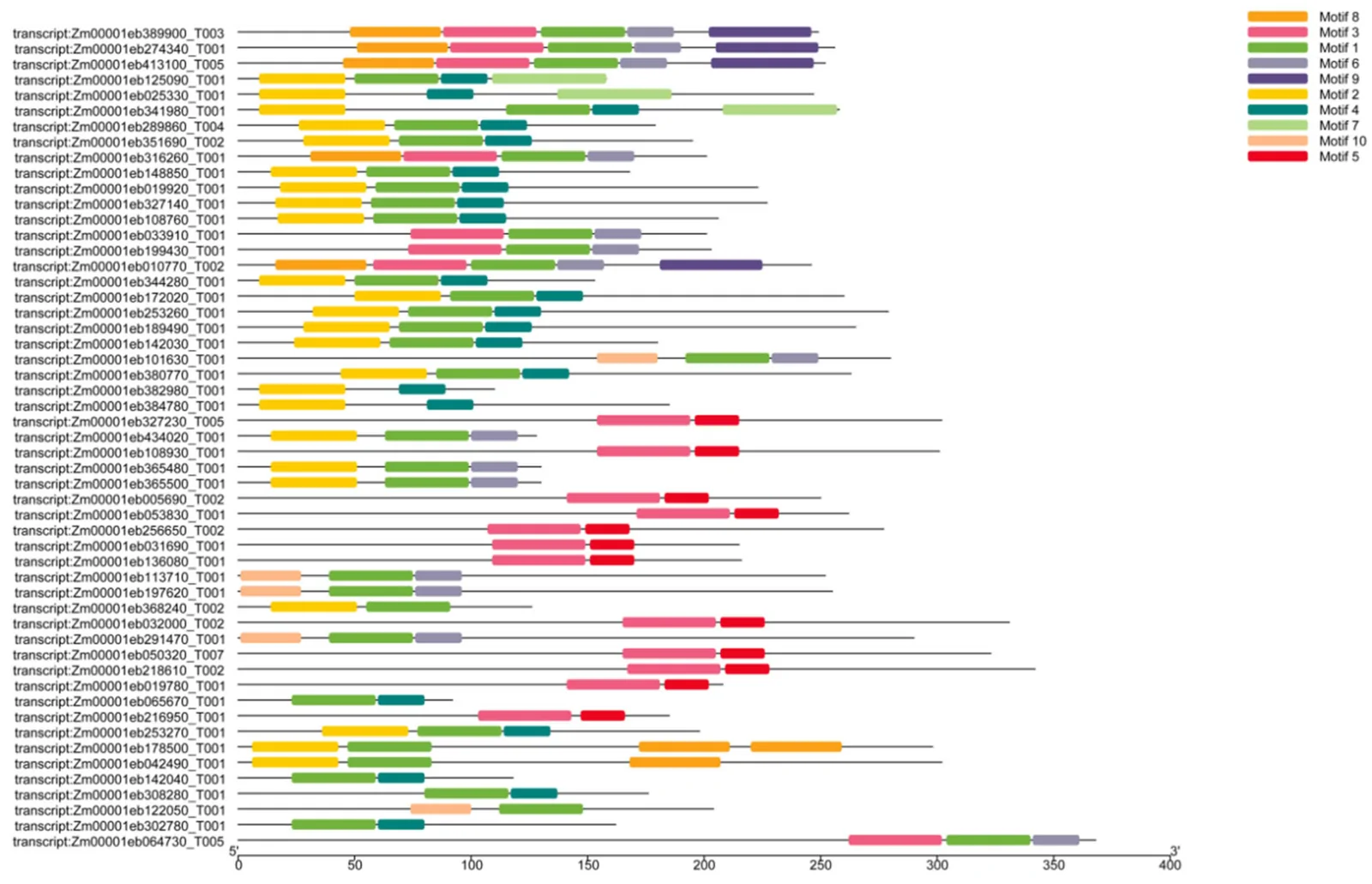
Conserved motif analysis of ZmNF-Ys. Supplementary Table S3 lists for NF-Y motifs sequence, size, and database functional annotation.

To investigate the conserved motif characteristics of the *ZmNF-Y* gene family, the MEME tool (https://meme-suite.org/meme/, accessed on 30 March 2025) was used to identify 10 classes of conserved motifs (Motif 1 to Motif 10) in the family members, and a visual analysis was performed (Figure 6, Supplementary Table S3). The results showed that among the conserved motifs of *ZmNF-Y* gene family, Motif 1, Motif 2, and Motif 4 are widely distributed; Motifs 1 and 4 overlap with the annotated conserved NF-Y functional domains, implying their potential functional significance. Motif 1 and Motif 4 are often adjacent to each other, forming the core functional domain of ZmNF-Y proteins, reflecting the high conservation of the core functional domain during evolution. The distribution of motifs within the family also shows notable differences, with significant variation in the number and arrangement of motifs among different ZmNF-Ys. For example, Zm00001eb389900 contains five distinct motifs, whereas Zm00001eb031690 contains only two motifs. Additionally, Motif 10 appears only in some genes, and the presence of such specific motifs may be closely related to functional differentiation. The composition and distribution of conserved motifs reveal both evolutionary conservation and functional divergence within the *ZmNF-Y* gene family. The presence of core conserved motifs underlies the basic functions of family members, while differences in specific motifs provide structural support for their involvement in diverse biological processes.

### 2.6. Cis-Acting Element Analysis of ZmNF-Ys

**Figure 7:**
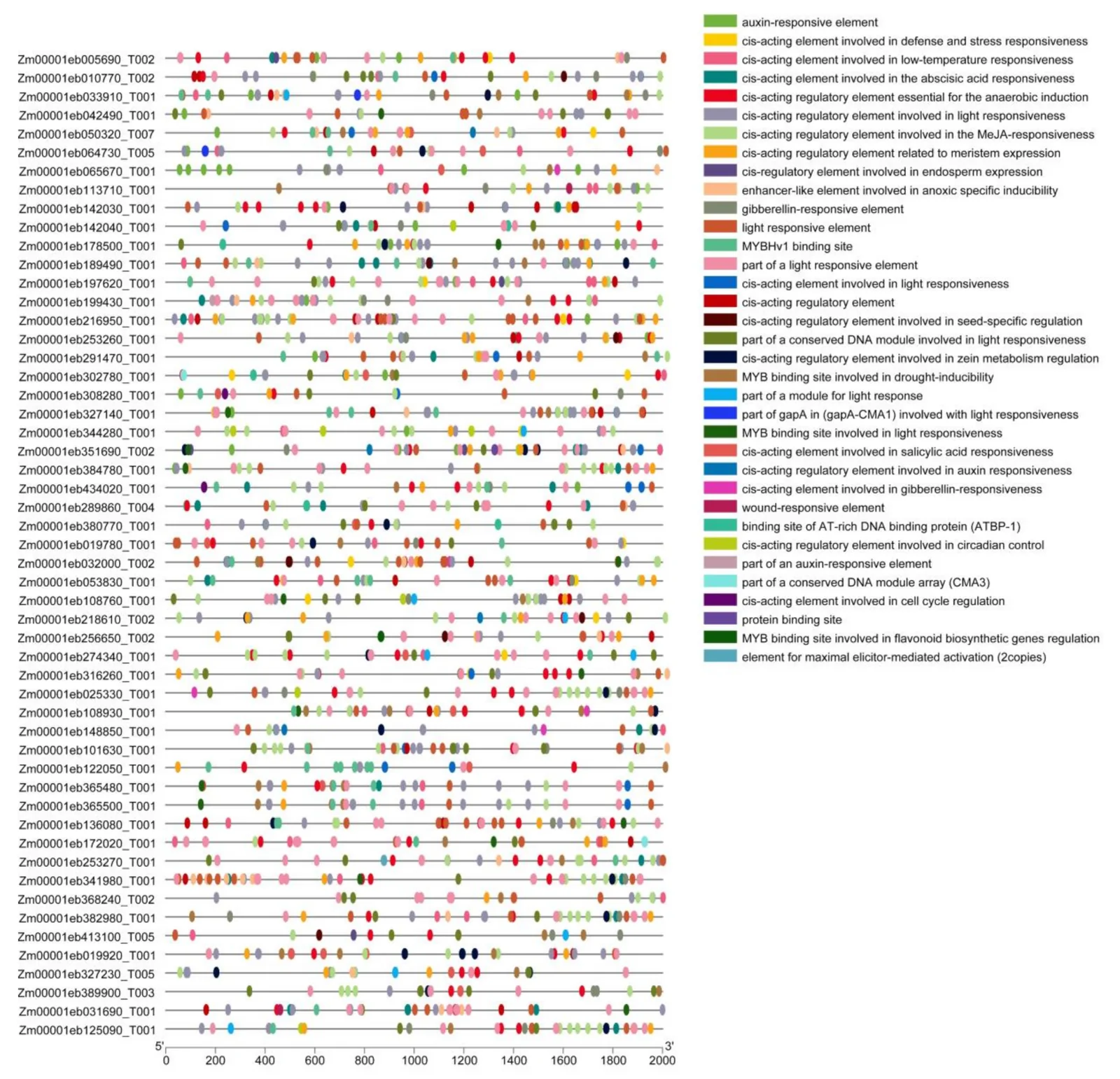
Cis-acting element prediction in the promoter regions of *ZmNF-Ys*. The key cis-acting regulatory elements are distributed in the 2000 bp region upstream of the *ZmNF-Y* genes, and different elements are shown in different colors. Supplementary Table S4 lists, for each *ZmNF-Y* gene: full element name, start-end position relative to the ATG codon, and copy number per promoter.

The promoter regions of *ZmNF-Y* gene family members contain abundant cis-acting elements, covering a wide range of types such as hormone response, abiotic stress response, growth and development regulation, and light response, indicating the complex regulatory functions of this gene family in maize growth, development, and environmental adaptation (Figure 7, Supplementary Table S4). In terms of element distribution, light-responsive elements are present in the promoters of most *ZmNF-Ys*, representing a widely distributed type of cis-acting element. This suggests that the expression of *ZmNF-Ys* may be commonly regulated by light signals and involved in photoperiod-mediated growth and development processes. Hormone-responsive elements also account for a significant proportion, with elements responding to auxin, abscisic acid, gibberellin, etc., distributed to varying degrees. This indicates that *ZmNF-Y* gene expression is co-regulated by multiple hormone signaling pathways and plays a role in hormone-mediated physiological processes. Abiotic stress-related elements, such as low-temperature response and drought-induced elements, are also present to varying degrees, suggesting that the *ZmNF-Y* gene family may be involved in stress responses to adversities like low temperature and drought, making it an important component of maize stress resistance regulatory networks. Additionally, growth and development-related elements were detected in the promoters of some genes, indicating that ZmNF-Ys also participate in the regulation of growth processes such as organ development in maize. Furthermore, the presence of binding sites for MYB, MYBHv1, and other transcription factors further reveals the complexity of the transcriptional hierarchy regulating *ZmNF-Y* gene expression.

### 2.7. Expression Analysis of the ZmNF-Y Family Genes Under Drought Stress

**Figure 8:**
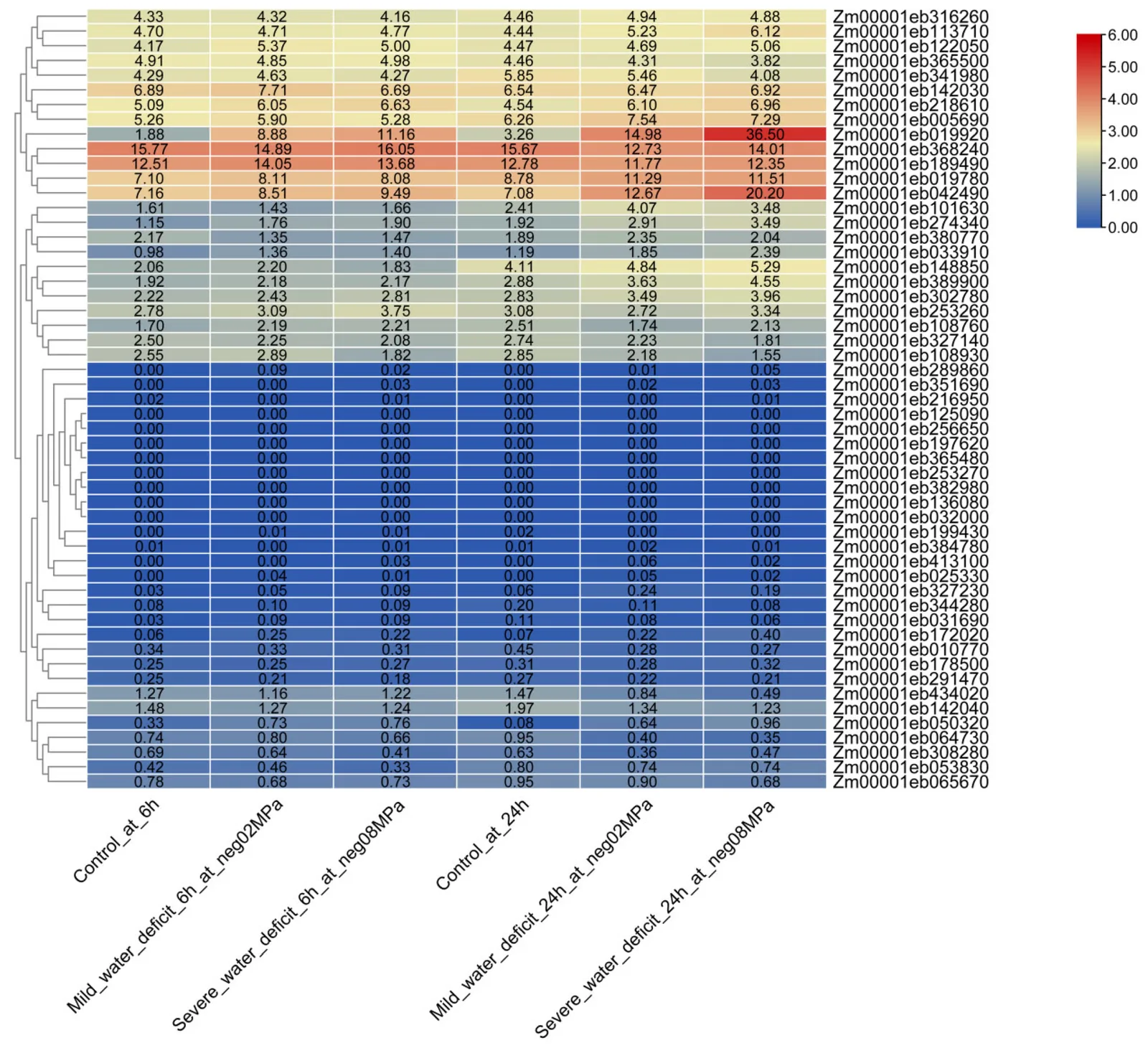
Heat map of differential expression of *ZmNF-Ys*. Drought treatment times are 6 and 24 h; −0.2 MPa represent moderate drought; −0.8 MPa represent severe drought. The heatmap scale bar (0.00–6.00) was generated using log_2_-transformed raw expression values. The color intensity reflected its log_2_-transformed magnitude.

To investigate the response of *ZmNF-Y* family genes to drought stress, a heatmap was constructed for the 53 genes (Figure 8). The results indicate that the expression levels of *ZmNF-Y* gene family members vary significantly under different degrees of drought stress and at different treatment time points. Based on the color gradients and numerical changes in the heatmap, the expression patterns can be classified into three categories: high expression, moderate expression, and low expression. The detailed analysis is as follows.

Characteristics of Stress Response in Highly Expressed Genes: Some *ZmNF-Y* genes exhibit strong transcriptional activation under drought stress. For example, the expression level of Zm00001eb019920 reached 36.50 under severe water deficit at 24 h, representing the peak value among all samples tested; Zm00001eb042490 showed a value of 20.20 under the same condition. These genes respond rapidly to drought-stress signals, and their expression increases sharply under severe water deficit, suggesting that they may serve as core response genes in maize drought-stress adaptation.

Expression Regulation Patterns of Moderately Expressed Genes: Some *ZmNF-Y* genes exhibit moderate expression levels under drought stress. For example, the expression level of Zm00001eb316260 was 4.46 in the control group at 24 h, increased to 4.94 under mild water deficit at 24 h, and slightly decreased to 4.88 under severe water deficit at 24 h. Zm00001eb218610 showed stable expression levels across all treatment groups, ranging between 4.50 and 7.00. This reflects a sustained adaptive response to drought stress.

Distribution Characteristics of Low-Expressed/Silent Genes: A large number of genes in the heatmap (such as Zm00001eb125090 and Zm00001eb256650) showed expression levels of 0.00 across all treatment groups, classifying them as silent genes. Another subset of genes (e.g., Zm00001eb384780) exhibited expression fluctuating only between 0.01 and 0.02, categorizing them as low-expressed genes. This group of genes was not significantly activated under drought stress, suggesting that they may not play a central regulatory role in the drought response pathways of maize.

In summary, within the *ZmNF-Y* gene family, the highly expressed responsive genes exhibit strong reactions to drought stress, while the functional roles of low-expressed genes require further investigation.

### 2.8. ZmNF-YA7 Positively Regulated Drought Tolerance in Maize

**Figure 9:**
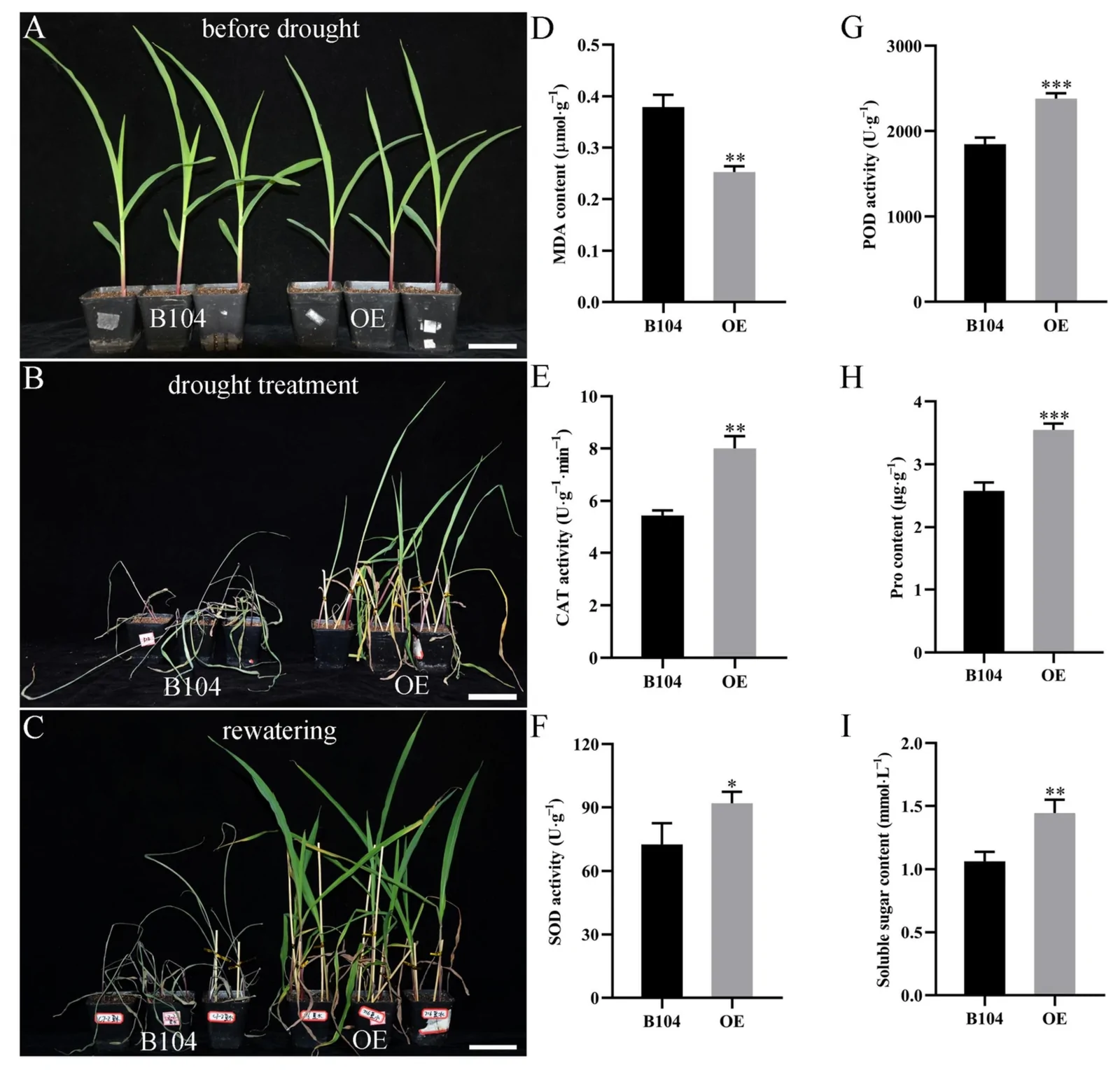
Soil drought treatment and physiological index analysis of B104 and OE. (**A**) Phenotype of B104 (control line) and OE (*ZmNF-YA7*-overexpressing in B104 transgenic plants) seedlings grown normally for 10 days. (**B**) Phenotype of seedlings with 18 days of drought treatment. (**C**) Phenotype of seedlings with 24 h after rewatering. (**D**) MDA content. (**E**) CAT activity. (**F**) SOD activity. (**G**) POD activity. (**H**) Pro content. (**I**) Soluble sugar content. The data represent the mean ± SD of three biological repeats, and the asterisks represent significant differences (* *p* < 0.05, ** *p* < 0.01, *** *p* < 0.001), n = 15. Scale bars = 8 cm in (**A**–**C**).

It is worth noting that gene Zm00001eb256650 (*ZmNF-YA7*) exhibited very low transcript abundance and was annotated as a “silent” gene under the given public drought-treatment conditions. Nevertheless, under laboratory-controlled soil drought conditions for 18 days, it was observed that the *ZmNF-YA7*-overexpressing transgenic plants (OE) exhibited enhanced drought tolerance compared to the control B104 (Figure 9A,B). Moreover, qRT-PCR results in our study demonstrated that *ZmNF-YA7* exhibited drought-responsive expression (Supplementary Figure S1). B104 showed significant leaf wilting, with the 1st to 3rd leaves completely desiccated (Figure 9B), while in OE plants, only the 1st leaf was desiccated, and the 2nd leaf displayed yellowing at the tip (Figure 9B). After 24 h of rewatering, the OE plants recovered rapidly and resumed normal growth, whereas B104 failed to recover (Figure 9C). These results indicate that under these drought-stress conditions, the *ZmNF-YA7*-OE transgenic plants possess superior drought tolerance compared to B104, and *ZmNF-YA7* positively regulates drought tolerance in maize B104.

Drought stress leads to the accumulation of ROS in plant tissues. Excessive ROS can damage sensitive biomacromolecules within cells, resulting in cellular injury and ultimately cell death. Malondialdehyde (MDA), one of the end products of membrane lipid peroxidation, serves as an indicator of the extent of lipid peroxidation and the degree of oxidative damage in plants. Measurement of MDA content in maize leaves after 7 days of drought treatment revealed a significantly lower MDA level in OE plants compared to B104 (Figure 9D).

Antioxidant enzymes, which play a crucial role in scavenging ROS, are key components of drought resistance mechanism in plants. Analysis of CAT, SOD, and POD activities in plants subjected to 7 days of drought showed that the activities of these antioxidant enzymes were significantly higher in OE than in B104 (Figure 9E–G). This suggests that OE plants possess a more efficient ROS-scavenging system, which mitigates oxidative damage and contributes to the enhanced drought tolerance. In addition, the contents of osmoprotectants such as proline and soluble sugars were significantly higher in OE compared to B104 (Figure 9H,I). Collectively, these results demonstrate that OE plants exhibit stronger drought tolerance than B104, indicating that ZmNF-YA7 positively regulated drought tolerance in maize.

### 2.9. ZmNF-YA7 Regulated the Growth of Vessels

Vessels, a component of the plant vascular system, serve as the main channels for transporting water and inorganic nutrients to the aerial parts. The formation of gas bubbles in vessels, which disrupts the continuity of the water column, is known as xylem embolism. This process can limit water loss under water stress, and thus embolism resistance is considered an indicator of plant drought tolerance. Embolism is influenced by vessel density and internal diameter: plants with lower vessel density and larger internal diameter typically exhibit higher hydraulic conductivity under normal conditions but are more prone to embolism under drought stress.

**Figure 10:**
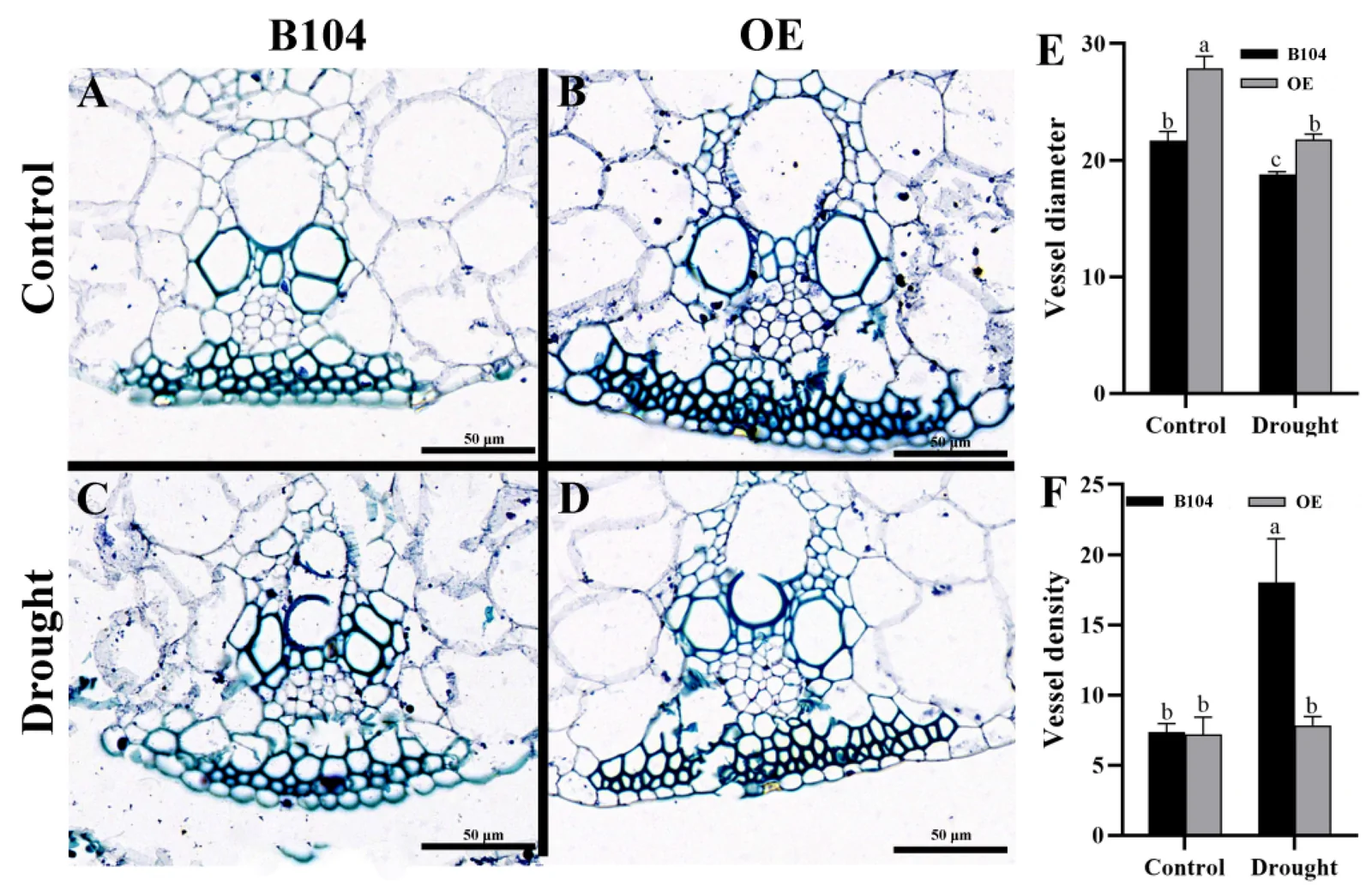
Microstructural analysis of cross-sections of young stems of B104 and OE. (**A**) Vascular bundles in normal growing B104 (control line) stem. (**B**) Vascular bundles in normal growing OE (*ZmNF-YA7*-overexpressing in B104 transgenic plants) stems. (**C**) Vascular bundles in B104 stem under drought conditions. (**D**) Vascular bundles in OE stem under drought conditions. (**E**) Statistics of vessel diameter. (**F**) Statistics of vessel density. Bars, 50 μm. The data represent the mean ± SD of three biological repeats, and the letters represent significant differences among groups (*p* < 0.05), n = 15.

Based on the above theoretical framework, we prepared and examined paraffin sections of transverse sections from the aerial parts of B104 and OE seedlings. Observations of xylem vessels in B104 and OE under both well-watered and drought conditions revealed that OE seedlings consistently exhibited larger vessel diameters than B104, regardless of water availability (Figure 10A–D). Statistical analysis of vessel internal diameters showed that both B104 and OE displayed reduced vessel diameters under drought compared to normal conditions; however, under drought stress, OE vessels remained significantly wider than those of B104 (Figure 10E). Regarding vessel density, no significant difference was observed between the two groups under normal conditions; however, under drought stress, vessel density differed markedly: OE showed significantly lower vessel density than B104 (Figure 10F).

These findings indicate that under drought conditions, OE seedlings develop xylem vessels with larger diameters and lower density compared to B104, which may render them more susceptible to embolism, thereby reducing excessive water loss and enhancing drought tolerance.

## 3. Discussion

### 3.1. The ZmNF-Y Gene Family Exhibits Evolutionary Conservation and Functional Divergence

In this study, we identified 53 *NF-Y* transcription factors in the maize genome. Consistent with findings in other species such as Arabidopsis and rice, the *ZmNF-Y* family is also categorized into three subfamilies: *ZmNF-YA*, *ZmNF-YB*, and *ZmNF-YC*, comprising 13, 25, and 15 members, respectively, indicating conservation of the family during evolution. The aa length, MW, and pI of ZmNF-Ys vary widely, with most members predicted to localize to the nucleus, aligning with their function as transcription factors. The identification of 48 syntenic gene pairs between maize and rice, far more than the 3 pairs identified with Arabidopsis, reflects a closer evolutionary relationship between maize and rice, which is consistent with their status as both being monocotyledonous plants. Intra-species collinearity analysis revealed numerous gene duplication events within maize, with chromosome 1 harboring the highest number of genes and gene pairs, suggesting that gene duplication, particularly on chromosome 1, has been a major driving force in the expansion and evolution of the *ZmNF-Y* family.

Analysis of gene structure and conserved motifs further reveals the balance between conservation and divergence within this family. While all members possess the core domain characteristics of the *NF-Y* family, significant differences exist in gene length, the number of introns/exons, and the composition and arrangement of conserved motifs. For instance, Motif 1 and Motif 4 are widely distributed and often adjacent, likely constituting the essential functional core. In contrast, the specific distribution of motifs like Motif 2 and Motif 10 may be associated with functional specialization among different subfamilies or individual members. This structural diversity provides a molecular basis for the functional diversification of *ZmNF-Y* genes.

### 3.2. The Promoter Cis-Element Profile Suggests That ZmNF-Y Genes Are Involved in Complex Regulatory Networks

Analysis of cis-acting elements in the promoters of *ZmNF-Ys* indicates that their expression is likely regulated by multiple endogenous and exogenous signals. The widespread presence of light-responsive elements suggests that *ZmNF-Ys* expression is commonly influenced by light signals, potentially linking their function to photomorphogenesis and the circadian clock. Additionally, the abundance of hormone-responsive elements (e.g., for auxin, abscisic acid, and gibberellin) and stress-responsive elements (e.g., for low temperature and drought) implies that ZmNF-Ys act as key integration nodes, mediating crosstalk between various hormonal pathways and abiotic stress responses to regulate maize growth, development, and environmental adaptation. This complex regulatory potential aligns with the diverse roles NF-Y transcription factors play in plants.

### 3.3. ZmNF-YA7 Positively Regulates Drought Tolerance in Maize by Modulating Physiological Responses and Xylem Development

Our study functionally characterizes *ZmNF-YA7* as a positive regulator of drought tolerance. Under drought stress, *ZmNF-YA7* overexpression (OE) lines exhibited milder leaf-wilting symptoms relative to B104, and showed better regrowth performance after rewatering. The physiological basis of this tolerance involves multiple mechanisms. First, OE plants accumulated less MDA, a marker of oxidative damage, indicating reduced membrane lipid peroxidation under drought. Second, OE plants maintained higher activities of key antioxidant enzymes (CAT, SOD, POD), enhancing their capacity to scavenge ROS and mitigate oxidative stress. Third, OE plants accumulated higher levels of osmolytes (proline and soluble sugars), which help to maintain cellular turgor and protect macromolecules. These coordinated physiological adjustments collectively contribute to the superior drought tolerance of OE plants.

A novel and significant finding of this study is that ZmNF-YA7 regulates xylem vessel anatomy. Under drought conditions, OE plants exhibited xylem vessels with larger diameters and lower density relative to B104. These anatomical results demonstrate that *ZmNF-YA7* overexpression remodels xylem hydraulic architecture—larger diameter and lower density—under drought. According to the hydraulic vulnerability theory, vessels with larger diameters are more prone to embolism (cavitation) under water stress, which can block water transport but also reduce water loss. We hypothesize that ZmNF-YA7 may influence drought tolerance not just at the biochemical level (antioxidants, osmolytes) but also at the structural level by modifying hydraulic architecture. Future hydraulic phenotyping, such as measurements of vulnerability curves and stem hydraulic conductivity, will be required to verify whether these anatomical alterations influence embolism formation and water conservation, as well as their functional contributions to drought tolerance.

### 3.4. Implications, Limitations, and Future Perspectives

This study provides a comprehensive genomic and preliminary functional analysis of the *ZmNF-Y* family in maize. We not only characterized the basic features and potential expression patterns of the ZmNF-Ys under drought but also demonstrated the crucial role of *ZmNF-YA7* in enhancing drought tolerance through integrated physiological and anatomical adaptations. The discovery that *ZmNF-YA7* modulates xylem development offers a new perspective on how transcription factors may regulate drought resistance by altering plant hydraulic traits.

However, this study has some limitations. First, the family-level drought-responsive expression data are retrieved from public MaizeGDB-qteller resources without our independent qPCR validation; related qPCR verification on representative genes will be performed in future work. Second, the expression analysis under drought was conducted at the transcriptional level; further protein-level validation and investigation of post-translational modifications are needed. Third, while we characterized *ZmNF-YA7*, the functions of the majority of ZmNF-Y members, especially those showing no response to drought, remain unknown. Fourth, the precise molecular mechanisms by which ZmNF-YA7 regulates antioxidant enzyme genes, osmoprotectant biosynthesis, and xylem development genes require further dissection, such as identifying its direct target genes through ChIP-seq, and screening vascular-related differentially expressed genes under drought conditions via transcriptome profiling. Fifth, the upstream regulatory relationship between *miR169s* and *ZmNF-YA7* in maize has not been validated in this work. Further experiments, including detecting the expression of the *miR169* family under drought stress and verifying their targeting interaction with *ZmNF-YA7*, will be performed in future investigations. Sixth, this observation is based on phenotypic images; quantitative survival-rate assays with larger plant populations will be required in future studies for solid statistical validation. In addition, physiological indices were only measured under drought-stress conditions while well-watered controls are absent; therefore, we cannot exclude constitutive physiological differences caused by *ZmNF-YA7* overexpression. Further experiments including normal-watering controls are needed to clarify genotype-treatment interactive effects.

Future research should focus on: (1) Functional and specificity analysis of different ZmNF-Y subfamilies using multiplex mutagenesis; (2) elucidating the transcriptional regulatory network downstream of ZmNF-YA7; (3) validating the role of ZmNF-YA7 in regulating hydraulic traits under field drought conditions and redundancy; and (4) exploring the potential of *ZmNF-YA7* and other drought-responsive *ZmNF-Y* genes in maize breeding programs aimed at improving drought tolerance.

## 4. Materials and Methods

### 4.1. Data Sources and the ZmNF-Y Gene Family Members Identification

The known 34 and 36 NF-Y family members for rice and Arabidopsis, respectively, were downloaded from the NCBI website (https://www.ncbi.nlm.nih.gov, accessed on 2 March 2025). We downloaded the whole genome files and genome annotation files for maize, Arabidopsis, and rice (GFF and GTF files for maize; GFF files for rice and Arabidopsis) from EnsemblPlants (https://plants.ensembl.org/index.html, accessed on 12 March 2025). The domain files (PF02045, PF00808) for *ZmNF-Ys* were downloaded from the Pfam website (https://pfam.xfam.org, accessed on 7 June 2025). The maize protein database was extracted using TBtools (v2.376), and the most representative transcripts were further retained. Subsequently, the DNA sequences encoding NF-Y were obtained through Blast (https://blast.ncbi.nlm.nih.gov/Blast.cgi, accessed on 9 March 2025) screening and HMMER (v3.3.2) screening. Finally, the protein sequences of the *ZmNF-Y* gene family members were extracted with TBtools (v2.376). Using the SMART database (https://smart.embl.de, accessed on 25 March 2025) and the NCBI-CDD database (https://www.ncbi.nlm.nih.gov/Structure/bwrpsb/bwrpsb.cgi, accessed on 13 June 2025), the obtained sequences were validated for domains. Following the removal of sequences lacking the relevant domains and redundant sequences, 53 sequences were identified.

### 4.2. Physicochemical Property Analysis of the ZmNF-Ys

The physicochemical properties of ZmNF-Ys were analyzed using TBtools (v2.376). The predicted parameters included the number of amino acids (aa), molecular weight (MW), theoretical isoelectric point (pI), instability index, and grand average of hydropathicity (GRAVY). Subcellular localization analysis of ZmNF-Ys was performed using Cell-PLoc 2.0 (http://www.csbio.sjtu.edu.cn/bioinf/Cell-PLoc-2/, accessed on 27 June 2025).

### 4.3. Phylogenetic Analysis and Chromosomal Localization of the ZmNF-Ys

To clarify the phylogenetic relationships and chromosomal distribution characteristics of ZmNF-Ys, we first performed a comparative analysis of NF-Y protein sequences from maize, Arabidopsis, and rice using MEGA12 software. A phylogenetic tree was constructed using the neighbor-joining method (with 1000 bootstrap replicates). Based on the positions of Arabidopsis and rice NF-Ys in the phylogenetic tree, the maize NF-Ys were classified into distinct subfamilies. The resulting tree was subsequently visualized and refined using the online tool iTOL (https://itol.embl.de, accessed on 1 June 2025). In parallel, the chromosomal locations of ZmNF-Ys were mapped using TBtools software (v2.376) in conjunction with the maize GFF annotation file.

### 4.4. Analysis of Collinearity in the ZmNF-Y Gene Family

The GFF files and whole-genome files of maize, rice, and Arabidopsis were analyzed using One-Step MCScanX-Super Fast plugin of TBtools (v2.376). Subsequently, TBtools (v2.376) was used to generate collinearity plots, including those illustrating intra-species collinearity within maize, as well as inter-species collinearity between maize and rice, and between maize and Arabidopsis.

### 4.5. Analysis of Gene Structure and Conserved Motifs of the ZmNF-Ys

The gene structures (including UTRs and CDS) of *ZmNF-Ys* were analyzed with TBtools (v2.376) and the maize GFF file, and a schematic diagram illustrating the gene structures was subsequently generated.

Conserved motif analysis was performed by analyzing the ZmNF-Y sequences using the MEME tool (https://meme-suite.org/meme/doc/meme.html, accessed on 30 March 2025). The maximum number of motifs was set to 10 in the MEME parameters, while all other parameters were kept at their default settings. Finally, the conserved motifs were visualized via TBtools (v2.376).

### 4.6. Analysis of Cis-Acting Elements of the ZmNF-Ys

Promoter sequences spanning 2000 bp upstream of the *ZmNF-Ys* were extracted from the maize GFF file and the whole genome file using TBtools (v2.376). The obtained promoter sequences were then submitted to the PlantCARE online analysis platform (https://bioinformatics.psb.ugent.be/webtools/plantcare/html/, accessed on 14 March 2025) for relevant analysis. Finally, visualization of the analysis results was performed using TBtools software (v2.376).

### 4.7. Differential Expression Analysis of the ZmNF-Ys Under Drought Stress

The specific expression data of the relevant genes were downloaded from the MaizeGDB-qteller database (https://qteller.maizegdb.org/, accessed on 23 March 2025), and an expression heatmap was subsequently generated using TBtools (v2.376).

### 4.8. Generation of Transgenic Maize

The CDS sequence of *ZmNF-YA7* was cloned and recombined into a modified pCambia3300 vector. ZmUbiquitin1 (ZmUbi) was used as the promoter to drive *ZmNF-YA7* expression. The *ZmNF-YA7*-overexpression construct was transformed into the maize inbred B104. The *ZmNF-YA7* sequence and primer inventory list are in Supplementary Table S5.

### 4.9. Drought Treatment and Growth Conditions

Maize seedlings were cultivated in a greenhouse under 16 h light/8 h dark photoperiod, 28 °C, 60–70% relative humidity, and light intensity of 400 μmol·m^−2^·s^−1^. Seeds were sown in plastic pots (diameter = 12 cm, height = 10 cm) filled with 200 g dry substrate (nutrient soil: vermiculite = 2:1, *v*/*v*), with three uniform seedlings retained per pot. Drought stress was initiated at the three-leaf stage. Watering was fully withheld for natural dehydration, and soil moisture was maintained at 15–18%.

Two drought experiments were performed. An 18-day continuous drought was applied for phenotypic and survival observation. A separate 7-day drought treatment was used for sampling to measure physiological indicators. Rewatering was conducted after 18-day continuous drought, and recovery phenotypes were recorded 24 h after rehydration. Each treatment included at least three biological replicates.

### 4.10. Measurement of MDA, Pro, and Soluble Sugar Contents and CAT, SOD, POD Activities

For each sample, 0.1 g of maize leaf tissue was weighed. We used the Malondialdehyde (MDA) Content Assay Kit (Catalog No.: BC0020), the Catalase (CAT) Activity Assay Kit (Catalog No.: BC0205), the Superoxide dismutase (SOD) Activity Assay Kit (Catalog No.: BC0170), the Peroxidase (POD) Activity Assay Kit (Catalog No.: BC0090), the Proline (Pro) Content Assay Kit (Catalog No.: BC0290), the Plant Soluble Sugar Content Assay Kit (Catalog No.: BC0030) from Solarbio(Beijing, China) for detection, respectively. All measurements were carried out strictly according to the manufacturer’s instructions. Three biological replicates were performed for each group. MDA content was expressed in μmol·g^−1^ FW; CAT activity was expressed in U·g^−1^·min^−1^; SOD and POD activities were expressed in U·g^−1^ FW; Pro content was expressed in μg·g^−1^ FW; and soluble sugar content was expressed in mmol·L^−1^.

### 4.11. Reproducibility and Statistical Analysis

Statistical analyses were indicated in each figure legend and performed using the program GraphPad Prism.v10. One set of statistical analyses were performed by two-way ANOVA analysis with Dunnett’s multiple comparisons test, with statistical significance indicated by the *p*-value (* *p* < 0.05, ** *p* < 0.01, *** *p* < 0.001). Other statistical analyses were performed by one-way ANOVA with Tukey’s multiple comparisons test, and two-way ANOVA analysis with Tukey’s multiple comparisons test, *p* < 0.05. All data are presented as mean ± SD. All experiments were repeated at least three times, and the exact sample size (n) is indicated in the corresponding figure legends.

### 4.12. Paraffin Sections

Fresh maize stem tissue were harvested and immediately immersed in a fixative solution. The fixed tissues were dehydrated using a graded ethanol series (70%, 80%, 90%, 95%, and 100%). The dehydrated tissues were cleared with xylene and embedded in molten paraffin wax. The paraffin blocks were sectioned. Finally, the sections were deparaffinized, rehydrated, stained, and mounted.

## Figures and Tables

**Table 1:** Physicochemical properties of the maize NF-Y family members.

Genes	Gene ID	Amino Acids	MW (Da)	pI	Instability Index	GRAVY	Subcellular Localization
*NF-YA1*	Zm00001eb216950	184	20,952.26	11.11	79.52	−0.611	Nucleus
*NF-YA2*	Zm00001eb218610	341	35,736.46	9.4	54.62	−0.381	Nucleus
*NF-YA3*	Zm00001eb108930	300	32,759.35	9.02	56.26	−0.829	Nucleus
*NF-YA4*	Zm00001eb031690	214	23,301.53	8.69	73.82	−1.087	Nucleus
*NF-YA5*	Zm00001eb005690	249	27,104.08	8.03	68.54	−0.841	Nucleus
*NF-YA6*	Zm00001eb053830	261	27,483.53	9.22	62.09	−0.617	Nucleus
*NF-YA7*	Zm00001eb256650	276	29,962.6	10.97	62.98	−0.59	Nucleus
*NF-YA8*	Zm00001eb064730	367	39,341.3	10.52	63.22	−1.305	Nucleus
*NF-YA9*	Zm00001eb019780	207	22,644.27	9.85	64.88	−0.784	Nucleus
*NF-YA10*	Zm00001eb050320	322	34,122.58	9.61	51.04	−0.508	Nucleus
*NF-YA11*	Zm00001eb327230	301	32,792.14	7.22	52.04	−0.872	Nucleus
*NF-YA12*	Zm00001eb136080	215	23,387.7	7.95	76.47	−1.025	Nucleus
*NF-YA13*	Zm00001eb032000	330	35,231.81	8.93	56.97	−0.461	Nucleus
*NF-YB1*	Zm00001eb380770	262	28,038.21	6.2	65.41	−0.652	Cytoplasm
*NF-YB2*	Zm00001eb344280	152	16,632.54	5.14	41.34	−0.594	Cytoplasm
*NF-YB3*	Zm00001eb351690	194	20,719.47	8.27	46.34	−0.452	Cytoplasm
*NF-YB4*	Zm00001eb327140	226	23,815.39	5.52	50.99	−0.646	Cytoplasm
*NF-YB5*	Zm00001eb108760	205	21,814.36	5.85	57.65	−0.666	Cytoplasm
*NF-YB6*	Zm00001eb302780	161	17,184.33	4.62	57.16	−0.266	Cytoplasm
*NF-YB7*	Zm00001eb178500	297	33,651.83	5.26	78.08	−0.876	Cytoplasm
*NF-YB8*	Zm00001eb125090	158	17,626.8	4.75	62.04	−0.455	Cytoplasm
*NF-YB9*	Zm00001eb172020	259	26,301.96	5.84	31.49	−0.456	Cytoplasm
*NF-YB10*	Zm00001eb148850	167	18,004.24	6.83	40.46	−0.678	Cytoplasm
*NF-YB11*	Zm00001eb142030	179	18,913	6.24	35.72	−0.622	Cytoplasm
*NF-YB12*	Zm00001eb253260	278	29,097.21	6.29	49.06	−0.522	Cytoplasm
*NF-YB13*	Zm00001eb019920	222	23,565.07	6.21	63.03	−0.846	Cytoplasm
*NF-YB14*	Zm00001eb065670	91	10,394.6	5.19	32.39	−0.807	Cytoplasm
*NF-YB15*	Zm00001eb289860	178	18,895.07	6.31	47.44	−0.649	Cytoplasm
*NF-YB16*	Zm00001eb142040	117	13,067.53	6.42	53.46	−0.859	Cytoplasm
*NF-YB17*	Zm00001eb042490	301	33,883.01	5.5	73.76	−0.898	Cytoplasm
*NF-YB18*	Zm00001eb341980	257	28,169.67	5.9	56.08	−0.621	Cytoplasm
*NF-YB19*	Zm00001eb368240	126	14,592.65	5.07	36.66	−0.304	Cytoplasm
*NF-YB20*	Zm00001eb025330	246	27,681.29	5.11	59.57	−0.379	Cytoplasm
*NF-YB21*	Zm00001eb253270	197	20,981.29	8.81	51.73	−0.695	Cytoplasm
*NF-YB22*	Zm00001eb189490	264	28,098.03	6.32	47.07	−0.633	Cytoplasm
*NF-YB23*	Zm00001eb308280	175	19,293.68	5.15	57.33	−0.754	Cytoplasm
*NF-YB24*	Zm00001eb382980	109	12,280.59	4.96	48.8	−1.004	Cytoplasm
*NF-YB25*	Zm00001eb384780	184	20,681.69	8.65	44.33	−0.332	Cytoplasm
*NF-YC1*	Zm00001eb101630	279	29,569.85	5.61	58.25	−0.052	Nucleus
*NF-YC2*	Zm00001eb389900	248	27,689.24	5.11	83.31	−0.541	Nucleus
*NF-YC3*	Zm00001eb316260	200	21,340.28	5.37	57.44	−0.206	Cytoplasm
*NF-YC4*	Zm00001eb291470	289	32,327.15	5.24	49.83	−0.83	Nucleus
*NF-YC5*	Zm00001eb274340	255	28,600.23	5.02	84.05	−0.581	Cytoplasm
*NF-YC6*	Zm00001eb434020	127	13,149.92	9.18	32.29	−0.069	Nucleus
*NF-YC7*	Zm00001eb199430	202	21,530.43	5.6	47.69	−0.209	Nucleus
*NF-YC8*	Zm00001eb113710	251	27,507.53	5.43	51.18	−0.799	Nucleus
*NF-YC9*	Zm00001eb197620	254	27,891.04	5.32	59.29	−0.774	Nucleus
*NF-YC10*	Zm00001eb365480	129	13,369.14	7.78	36.88	−0.076	Nucleus
*NF-YC11*	Zm00001eb365500	129	13,337.14	8.57	39.45	−0.078	Nucleus
*NF-YC12*	Zm00001eb033910	200	21,549.6	5.65	57.8	−0.309	Nucleus
*NF-YC13*	Zm00001eb413100	251	28,028.71	5.21	70.15	−0.575	Cytoplasm
*NF-YC14*	Zm00001eb010770	245	25,989.28	5.2	64.03	−0.33	Nucleus
*NF-YC15*	Zm00001eb122050	203	21,295.62	4.89	62.19	−0.553	Nucleus

## Data Availability

All data supporting the findings of this study are available within the paper.

## Supplementary Materials

The following supporting information can be downloaded at: https://www.mdpi.com/article/10.3390/ijms27188319/s1.
