# Supplementary material for: Genome-Wide Identification of the ZmNF-Y Transcription Factor Family in Maize and Functional Characterization of ZmNF-YA7 in Drought Tolerance
Source: Int J Mol Sci. 2026 Sep 18;27(18):8319. doi: 10.3390/ijms27188319 (PMC13607155; doi:10.3390/ijms27188319)
Supplement: Supplementary file 1 [file ijms-27-08319-s001.zip › Supplementary Figure S1.pdf]

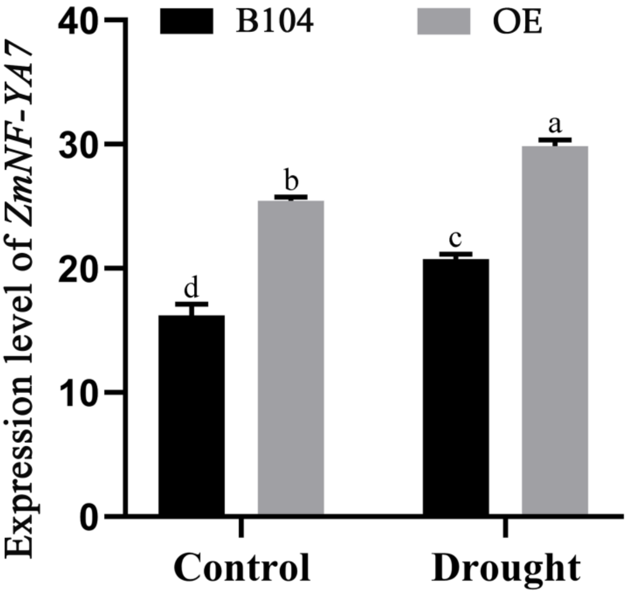

Supplementary Figure S1. As shown, *ZmNF-YA7* transcript levels were practically identical in OE lines and in WT under normal conditions, and were markedly induced by drought stress, whereas control lines showed no such induction. Data are shown as means ± SD, n = 3. Significant differences among all plants are indicated with different letters, $P < 0.05$.
